# Post-thaw storage of sperm as a tool towards revealing paternal-effect-associated genes in Eurasian perch, *Perca fluviatilis*

**DOI:** 10.1186/s12864-026-12648-4

**Published:** 2026-02-20

**Authors:** Abhipsa Panda, Sylwia Wałdowska, Katarzyna Palińska-Żarska, Rossella Debernardis, Joanna Nynca, Rafał Rożyński, Anna M. Majewska, Jan P. Jastrzębski, Daniel Żarski

**Affiliations:** 1 Team of Reproduction and Development in Fish, InLife Institute of Animal Reproduction and Food Research, Polish Academy of Sciences, Olsztyn, Poland; 2 Department of Ichthyology, Hydrobiology and Aquatic Ecology, National Inland Fisheries Research Institute, Olsztyn, Poland; 3 Department of Salmonid Research, National Inland Fisheries Research Institute, Rutki, Poland; 4 Team of Gamete Biology, InLife Institute of Animal Reproduction and Food Research, Polish Academy of Sciences, Olsztyn, Poland; 5 Department of Plant Physiology, Genetics, and Biotechnology, Faculty of Biology and Biotechnology, University of Warmia and Mazury, Olsztyn, Poland

**Keywords:** Eurasian perch larvae, Paternal-effect-associated genes, Transcriptomics, Post-thaw storage of milt

## Abstract

**Background:**

The influence of paternity on progeny quality, particularly during early developmental stages, has long been underappreciated. However, altered sperm phenotypes are increasingly recognized as effective tools for identifying paternal-effect-associated genes (PEAGs), whose expression in the progeny is influenced by genetic or non-genetic factors carried by the sperm. This study investigated the impact of post-thaw sperm storage (PTS) as a stressor to verify its effect on larval performance in common garden rearing trial and to reveal PEAGs in Eurasian perch (*Perca fluviatilis*) progeny. In vitro fertilizations were performed using cryopreserved sperm that was either used immediately after thawing (0 min; CON) or after 30 min of post-thaw storage at 4 °C.

**Results:**

Despite a marked decline in sperm motility during PTS, fertilization success remained unaffected, allowing the use of PTS to study its effect on progeny phenotype. Notably, larvae from the PTS group exhibited significantly higher mortality starting from 9 days post hatch, indicating strong paternal influences on early larval viability. Transcriptomic profiling of larvae at the mouth-opening stage, selected to minimize rearing-induced variation, identified 41 differentially expressed genes (DEGs), many linked to immune regulatory pathways. This suggests that paternal inputs may shape larval immune function, potentially contributing to observed mortality differences. Among the DEGs, several genes, *mfap4*, *gimap*, *hlag*, *pigr*, *neo1*, and *pde6g*, emerged as strong candidate PEAGs.

**Conclusions:**

This study shows that even a brief, 30-minute PTS not only reduces sperm motility but also imprints lasting effects on progeny performance and survival. By selectively shaping the pool of functional sperm, PTS acts as an additional layer of selection, enriching for cells with specific traits and offering a powerful, controlled system for studying non-genetic inheritance factors and identifying PEAGs in fish. Transcriptomic analysis uncovered a deeper dimension to this process, revealing that maternal identity can amplify or buffer paternal contributions which serves as evidence of a complex parental interplay that influences early development. In effect, this study provides a robust experimental model based on controlled, paired fertilization design together with transcriptomic profiling of the offspring to identify novel PEAGs and reveal molecular consequences of paternal variation induced by post-thaw storage.

**Supplementary Information:**

The online version contains supplementary material available at 10.1186/s12864-026-12648-4.

## Background

Reproduction in fish is a multifaceted process where maternal and paternal contributions jointly determine offspring phenotype. The maternal contribution in fish, encompassing both genetic factors (such as inherited alleles) and non-genetic factors (including RNA, proteins, and lipids deposited in the eggs), is known to control development at least up to zygotic genome activation (ZGA), thereby determining key aspects of the embryo’s phenotype and initial developmental trajectory [1, 2]. In contrast, paternal influences have long been underemphasized, in part because aquaculture practices traditionally prioritized female quality and often pooled sperm, reducing attention to male-specific contributions. From a scientific perspective, paternal effects were also difficult to resolve, as sperm contribute minimal cytoplasmic material and disentangling subtle sperm-borne influences from dominant maternal effects has been technically challenging [3, 4]. However, over several decades, controlled breeding experiments have enabled to provide plenty of evidences that paternal identity contributes significantly to variation in early life-history traits across multiple fish species. Significant paternal effects have been reported across a range of early developmental traits, including embryo survival, hatching, larval morphology, early growth, and even larval size at hatch. These effects have been observed across multiple fish species, such as haddock (*Melanogrammus aeglefinus*; [5, 6]), winter flounder (*Pseudopleuronectes americanus*; [7, 8]), sole (*Solea solea* L.) and herring (*Clupea harengus*; [9]), cod (*Gadus morhua*; [10–12]), as well as brown trout (*Salmo trutta* L.; [13]), rainbow trout (*Oncorhynchus mykiss*; [14]), masu salmon (*Oncorhynchus masou*; [15]), Atlantic salmon (*Salmo salar*; [16]), eel (*Anguilla anguilla*; [17]), catfish (*Ictalurus punctatus*; [18]), ide (*Leuciscus idus*) and Northern pike (*Esox lucius*; [19]). Furthermore, sperm physiological traits (including ATP content, motility duration, and activation patterns) have been directly linked to offspring developmental outcomes, as demonstrated in Atlantic herring [20]. Together, these studies clearly show that paternal identity and sperm phenotype represent biologically meaningful determinants of early developmental trajectories in fishes. However, despite this extensive body of work, most studies have focused on phenotypic or zootechnical outcomes, whereas the underlying molecular consequences of paternal effects remain considerably understudied.

Emerging research has illuminated that paternal effects extend to epigenetic modifications of sperm, controlled by paternal conditions (like stress, age, nutrition), can alter offspring traits through non-genetic means [21]. Epigenetic modifications, such as DNA methylation or histone modifications, along with non-coding RNAs present in sperm are transmitted as molecules, recognized as carriers of non-genetic information. These modifications can influence gene expression in the developing progeny, a phenomenon often referred to as non-genetic inheritance (NGI) [3]. In zebrafish (*Danio rerio*), paternal stress exposure altered the sperm small RNA landscape, particularly microRNAs and PIWI-interacting RNAs, which resulted in larvae showing attenuated behavioral and stress responses, indicating a direct influence of environmental conditions on sperm-mediated inheritance [22]. Also, the paternal methylation pattern serves as a template, while the maternal methylation pattern is largely reprogrammed based on paternal epigenetic information in zebrafish progeny [23, 24]. Thus, a growing body of evidence suggests that paternal contributions may modulate developmental trajectories and adaptive responses also on a molecular level [4, 25]. Yet, the scarcity of studies addressing these effects highlight a significant gap in our understanding, one that is critical for both evolutionary biology and applied fields like aquaculture [19].

The first step towards understanding paternally-controlled traits is the identification of genes whose expression in the offspring reflects paternal effects, defined as father-mediated influences on offspring phenotypes, including molecular traits, that arise via non-genetic mechanisms rather than allele transmission [26]. Such genes are hereafter referred to as paternal-effect-associated genes (PEAGs). Recently, we have found that cryopreservation, which is a vital technique for long-term storage of sperm, is an ideal tool for the identification of such genes in Eurasian perch (*Perca fluviatilis*) [27], a freshwater teleost fish species of increasing aquaculture relevance. With well-established protocols for reproduction and larviculture, the species suits well for molecular studies aiming domestication, adaptability to aquaculture conditions, circadian rhythm and reproductive biology [28, 29]. Thus, combined with available genomic resources [2, 30] and larval rearing protocols using Eurasian perch as a model species offers a robust scientific approach for investigating paternal-effect-associated genes in progeny. Working on this fish model, we have demonstrated that identified PEAGs are responsible for the early development of the fish visual system. These findings are linked to the fact that cryopreservation presents specific challenges for sperm cells, leading to structural, molecular, and functional changes within the spermatozoa population and therefore reducing motility and fertilizing ability among cryo-sensitive spermatozoa [31–34]. Consequently, this technique exerts additional pressure on sperm cells, where only the most robust (cryo-resistant) cells retain their fertilizing capacity, a phenomenon we term “cryo-selection”, and having direct contribution to the larval phenotype [27, 35–37]. Thus, our study showed that cryo-selection is crucial in shaping progeny phenotype, largely related to non-genetic factors (e.g., epigenetic states) conveyed by positively cryo-selected (cryo-resistant) sperm cells. And this was noticeable even with well-standardized cryopreservation protocol for Eurasian perch milt, ensuring high motility and fertilizing ability after thawing [38]. To add on to this, we were focusing on freshly hatched larvae transcriptome and their further performance, also seen as early life history of the progeny, which provides a critical window to assess how paternal effects manifest following exposure of the sperm to normal and stressed conditions [39]. Larval phase is relatively short, but important period in fish ontogeny, where the interplay between genetic inheritance, NGI and environmental factors is highly dynamic [40]. Therefore, examining larval performance along with molecular characterization of their entire body, has been pointed out as a very robust and valuable approach towards exploring complexity of the phenotype stemming from parental contributions [2].

Post-thaw storage (PTS), defined as the period during which thawed sperm is stored before use for fertilization, is a technically unavoidable and often overlooked component of cryopreservation workflows in aquaculture and research. It has been evidenced that delays between thawing and fertilization can introduce additional physiological stress, accelerate sperm deterioration, and potentially influence which spermatozoa ultimately succeed in fertilizing the eggs. Although cryopreservation protocols have been investigated for Eurasian perch (see [38, 41, 42]), post-thaw sperm quality still declines, and further drastic reduction in motility occurs during post-thaw storage (PTS; from ~ 70 immediately after thawing to ~ 35% after 30 min of storage), a phenomenon characteristic of this species. Therefore, it can be suggested that cryopreserved perch milt should be used for fertilization of eggs immediately (within just few min) after thawing. Thus, PTS period can be considered as an additional selection factor for cryo-resistant spermatozoa adding supplementary challenge test to the sperm. In this context, previous studies focused only on sperm motility [38, 41, 42], the main mechanistic pathways by which PTS affects progeny remain unclear. It is still to be elucidated whether PTS imposes a selective pressure favoring more robust sperm, causing sub-lethal sperm damage that manifests in larvae, or involves a combination of both mechanisms. Currently there is no information regarding the effect of PTS on fertilization ability of Eurasian perch spermatozoa as well as its long-term effects on subsequent developmental stages, specifically larval performance and their molecular portrait. Together, insights from larval performance and molecular profiling provide a more complete understanding of how PTS influences offspring quality, offering valuable information for the use of cryopreserved sperm in both basic biological research and aquaculture breeding.

Consequently, we hypothesized that post-thaw storage acts as an additional stressor that selectively alters the functional composition of spermatozoa contributing to fertilization, resulting in measurable differences in progeny phenotype and early-life transcriptomic profiles, including the emergence of paternal-effect-associated genes.

## Materials and methods

### Experimental design

**Fig. 1:**
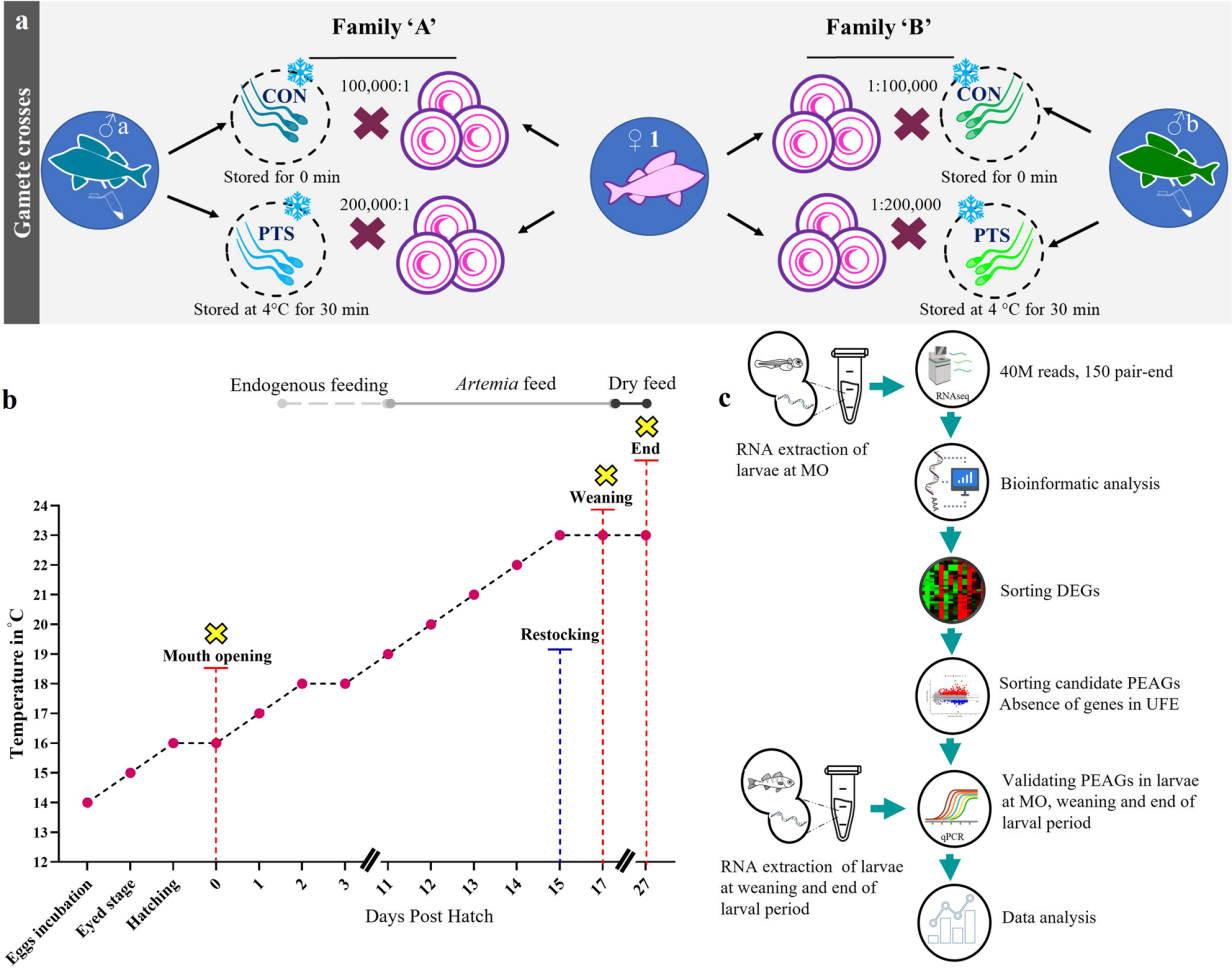
Experimental design and protocol followed. **a** Creation of families, where one female (exemplarily female 1) was crossed with two males (exemplarily male a and b here, created families A and B, respectively), with groups as 0 min post-thaw stored (control group; CON) with the sperm: egg ratio 100,000:1 and for the 30 min post-thaw stored (PTS) group being 200,000:1. This gamete crossing operations were done with 3 females and 6 males in the same format. **b** Feeding schedule and temperature regime followed for the larval rearing. The yellow crosses represent sampling points. **c** Experimental workflow summarizing processing of RNA and data obtained from larvae at various sampling points. MO – Mouth opening; DEGs – differentially expressed genes; PEAGs – Paternal-effect-associated genes; UFE – Unfertilized eggs

To investigate the role of paternal contributions in early development, this study aimed to identify PEAGs by applying PTS as an additional stressor in a cryo-selection framework. In this study, we performed in vitro fertilizations using cryopreserved sperm that was either used immediately after thawing (control group, CON) or stored post-thaw for 30 min at 4 °C (PTS group). A total of twelve families were produced. Eggs from each female were divided into four equal portions (~ 25 g each), ensuring equal distribution of eggs for fertilization trials conducted in triplicate (Fig. 1a). Two portions were fertilized with sperm from the same male, one with CON sperm and the other with PTS sperm. The remaining two portions were fertilized with CON and PTS sperm from a different male (Fig. 1a). This procedure was repeated three times, resulting in three females being crossed with six different males in total. Larviculture was subsequently carried out to monitor zootechnical parameters and collect samples for transcriptomic analysis, enabling a comparison between the CON and PTS groups.

### Broodstock management and controlled reproduction

All the manipulation with fish (hormonal injection, gametes collection) were performed under the anesthesia in MS-222 (150 mg L⁻¹; Argent, USA).

### Reproduction through males: sperm collection, quality analysis and cryopreservation

For the study, six cultured males (physiological data in Additional file, sheet 1) were used, that were reared in the Department of Salmonid Research of the National Inland Fisheries Research Institute (NIFRI) in Rutki (North Poland) and were overwintered for three months under natural photothermal conditions in a flow-through system supplied with riverine water. Fish were fed with commercial compound feed (AllerAqua Gold). At the early phase of the spawning season, where males were slightly spermiating (a small drop of sperm was possible to be spotted after gentle massage of their abdomen), they were transported in oxygenated water-filled plastic bags, to laboratories of NIFRI in Olsztyn (North-Eastern Poland), where they were placed in recirculating aquaculture system (RAS) at a constant temperature (12 °C) and photoperiod (14 L:10D). Right after arrival, fish were injected with salmon gonadoliberin analogue (sGnRHa, BACHEM, Switzerland) at a dose of 50 µg kg⁻¹ [43] to promote spermiation.

**Fig. 2:**
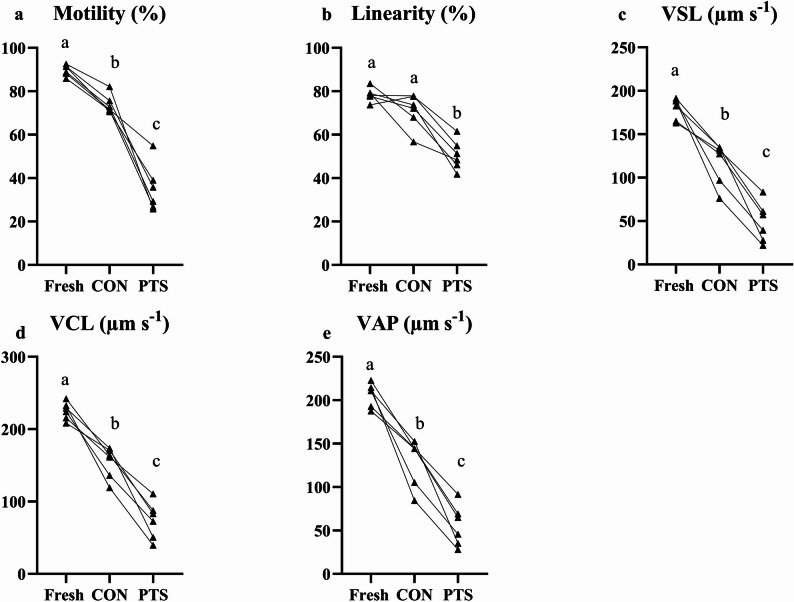
Sperm motility parameters [motility (**a**), linearity (**b**), VSL (**c**), VCL (**d**) and VAP (**e**)] in fresh, CON and PTS groups. Different superscripts indicate significant differences (*p* < 0 .05) between Fresh, CON and PTS sperm. 0 min post-thaw (control group; CON) and 30 min post thaw-stored (PTS group) sperm

Milt was collected after 10 days of hormonal stimulation using gentle abdominal pressure and a catheter (Galmed, Poland) to prevent contamination with urine or blood [44]. Samples were stored on ice immediately after collection. Parameters like sperm motility (Fig. 2a), concentration and viability (with the use of NucleoCounter SP-100, Chemometec, Denmark) of fresh milt were assessed ensuring that good quality of milt was used for cryopreservation [41] (see Additional file, sheet 1). Then, milt was cryopreserved and its motility was further evaluated at 0 and 30 min after thawing using a two-step activation process. Initially, fresh milt was diluted 1:50 and frozen/thawed milt 1:5 in an immobilizing solution [45] (150 mM NaCl, 5 mM KCl, 1 mM MgSO₄ × 7 H₂O, 1 mM CaCl₂ × 2 H₂O, 20 mM Tris, pH 8.0). Activation was achieved by further dilution (1:20) in an activating solution of 75 mM NaCl, 2 mM KCl, 1 mM MgSO₄ × 7 H₂O, 1 mM CaCl₂ × 2 H₂O, 20 mM Tris, pH 8.0, supplemented with 0.5% bovine serum albumin [41]. Various sperm parameters like motility (MOT, %), linearity (LIN, %), curvilinear velocity (VCL, µm s⁻¹), average path velocity (VAP, µm s⁻¹), and straight-line velocity (VSL, µm s⁻¹), were evaluated for both fresh, cryopreserved and PTS sperm using the computer-assisted sperm assessment (CASA; CEROS II system -Hamilton-Thorne, USA) system.

Cryopreservation of milt was conducted following the standardized protocol [38] consisting of a final concentration of 0.3 M glucose, 7.5% methanol and 25 mM KCl with a spermatozoa concentration of 3 × 10⁹/ml. This procedure ensures that each straw contains an equal concentration of spermatozoa in cryopreserved samples of each individual. The milt diluted with extender was loaded into 0.5 mL plastic straws (IMV Technologies, France). Cryopreservation was performed using a commercially available unit consisting of a styrofoam box (external dimensions: L 64 × W 41 × H 28 cm) with an inner stainless-steel container and lid (inner dimensions: L 50 × W 30 × H 20 cm), a loading device, and a floating rack for 90 straws (Ref.: 15043/0736, Minitübe GmbH, Germany). The cryopreservation process was carried out by placing the straws on the floating rack positioned 3 cm above the surface of liquid nitrogen (LN₂) in a cryopreservation unit containing approximately 7 L of LN₂. The straws were exposed to nitrogen vapor for 5 min. The straws were then transferred to liquid nitrogen for their further use, aimed at fertilization of eggs [46].

### Reproduction through females: eggs collection

For the experiment, three females caught using fyke nets from Mikołajskie lake (North-Eastern Poland) were used (physiological data in Additional file, Sheet 1). They were transported to NIFRI in Olsztyn in the same way as the males. Females were maintained in a RAS with controlled photoperiod 14 L:10D and water temperature of 12 °C (± 0.1 °C) until ovulation. Wild fish do not feed under hatchery conditions prior to reproduction operation [47]. Females were stimulated with the same dose of sGnRHa hormone was provided to them as males (50 µg kg⁻¹). Oocyte maturation was evaluated by catheterizing females, exposing collected small sample of oocytes to Serra’s clarifying solution (ethanol, formalin, and glacial acetic acid in a 6:3:1 ratio), and assessing their developmental stage microscopically [48]. At ovulation, the fish were anesthetized, and eggs were stripped into clean, dry beakers.

Eurasian perch eggs are released as a cylindroconical gelatinous ribbon, in which individual eggs are embedded within a thick gelatinous matrix and connected to one another. Freshly stripped egg ribbons were thus gently divided by hand into larger segments (26.3 ± 1.4 g) by tearing the gelatinous structure along its natural continuity. Fertilization was performed on these larger ribbon segments. The sperm-to-egg ratio was standardized using egg mass as a proxy for egg number, with 1 g of eggs used as a calculation unit. Based on the estimated egg number per gram (~ 500 eggs per 1 g), the corresponding sperm volume was calculated and proportionally adjusted for each egg ribbon segment prior to fertilization.

### Fertilization trials

Cryopreserved sperm straws were thawed at 40 °C for 10 s in a water bath, and then milt was poured into an Eppendorf, kept at 4 °C for 30 min (PTS). At the end of this storage period, the same thawing procedure was used for freshly thawed milt (CON) from the same male without providing them a waiting period, enabling fertilizations for both groups simultaneously. For the fertilization of eggs in CON group egg to sperm ratio was 1:100,000, whereas for PTS, 1: 200,000; in order to compensate for the number of motile spermatozoa, which decreased after 30 min of PTS. For fertilization, 16.7 µl and 33.4 µl of cryopreserved milt were used per each 1 gram of eggs for the CON and PTS groups, respectively. Moreover, adjusting the sperm-egg ratio allowed us to control for this dosage effect and ensure that any differences observed between groups reflected sperm quality rather than sperm quantity. Consequently, our experimental design was based on doubling the number of motile spermatozoa in the PTS group, which enables us to use the same nominal number of sperm for both treatments. Because the objective of the study was not to test how reduced sperm motility affects fertilization, but rather how differently handled sperm influence offspring phenotype and transcriptomic profiles, it was essential to equalize the number of motile sperm available to fertilize the eggs. Just before fertilization, eggs were preactivated in Woynarovich solution (3 g Urea, 4 g NaCl in 1 L ddH_2_O; given in the ratio of 1:5, eggs: solution) for 30 s before sperm was introduced [49]. After 10 min, excess sperm and debris were washed away with hatchery water.

### Eggs incubation and larval hatching

The eggs were incubated in 15 L tanks with black walls and upper water inflow, which functioned within the same RAS. The eggs were spread on mesh (diameter of around 3 mm) and kept in water at a temperature of 14 °C. A piece of the egg ribbon (~ 100 embryos) was observed under the microscope (Leica, Germany) at the different developmental stages after fertilization, including blastula, epiboly, neurula, optic cup, tail detachment, up to full eye pigmentation. This was followed by the hatching larvae count and the deformed larvae count. The photoperiod during incubation of all embryos and subsequent larval rearing was maintained at 24 L:0D (1500 lx, measured at the water surface). Embryos were kept at the initial temperature until reaching the eyed-egg stage, at which point it was raised to 15 °C. Following the observation of the first hatched larvae, the temperature was further increased to 16 °C. To maintain synchronous hatching, the larvae were hatched manually. This was done by transferring the egg ribbons to bowls with water from the rearing tanks and stirring gently [29]. This operation was repeated a few times until most of the larvae hatched. The day of hatching was considered as 0-day post-hatching (DPH). After hatching, the larvae were left undisturbed for 24 h. On 1 DPH, the deformed larvae (%) were counted in the subsamples and discarded as they would not survive. On 2 DPH, larvae were counted volumetrically and distributed into tanks at a density of 1500 larvae per tank. Each biological replicate from CON and PTS group were reared separately in triplicate (in total in 36 tanks).

### Larval rearing

Larvae were reared under standardized conditions [29] (as shown on Fig. 1b). Feeding with micro Artemia cysts (SF origin) began at 4 DPH (at a prey density of 13 nauplii per mL supplied twice a day). From 4 DPH onwards, feeding success was assessed daily by randomly sampling approximately 100 larvae from each tank and examining their stomach contents under a stereoscopic microscope (Leica, Germany) to check for the presence of food. This continued until 10 DPH, when all non-feeding larvae had died. Starting at 4 DPH, swim bladder inflation effectiveness (SBIE, %) was also counted in the same way as feeding rates and was recorded until 10 DPH. After this time, no increase in the number of larvae with filled swim bladders was observed. The larvae were fed with standard-size Artemia cysts at 260,000 nauplii per gram (GSL origin) since 8 DPH (at a prey density of 10 nauplii per mL supplied three times a day). In addition, from 12 DPH, dead larvae were examined under the microscope to evaluate the type I cannibalism (when the prey is partially ingested) [50]. Two days before weaning (15 DPH), larvae from each family were counted manually and restocked at a density of 400 larvae/tank. This was done to ensure the same number of larvae in each tank, which varied due to the different mortality rates observed in some families. After weaning (that took place at 17 DPH), larvae were fed exclusively with dry feed (Perla Larva Proactive, Skretting, Norway) three times a day, sprinkling it into each tank in small amounts for ~ 15 min each time [29, 51]. Mortality rates (%) were counted twice each day while the tanks were being cleaned. Oxygen levels in the tanks were checked daily using an OxyGuard^®^ Polaris (Denmark) and never dropped below 80% saturation. Ammonia and nitrite concentrations were measured every two days using a DR1900 Portable Spectrophotometer (Hach^®^, USA) and remained below 0.02 mg L^–1^. The experiment ended on 27 DPH when more than 50% of the larvae showed no fin fold; therefore, more than half of the fish had already finished the larval period [29].

### Sampling points

Larvae were sampled at three key time points (Fig. 1b) for RNA extraction, as well as for measurements of total length (TL, mm) and wet body weight (WBW, mg). These stages included: (i) mouth opening (MO; 0 DPH), when the progeny have undergone least human intervention, (ii) at weaning as they transition to commercial compound feed (17 DPH) [52], and (iii) the end of the larval period (27 DPH). At each time point, 30 larvae per family (10 individuals from each of three replicate tanks) were collected for morphometric assessment. To measure TL and WBW, larvae were first anesthetized. For TL measurement, anesthetized individuals were photographed under a stereomicroscope (Leica, Germany). WBW was determined using a precision balance by placing the larvae on a nylon mesh (approx. 200 μm) and gently blotting away excess moisture with filter paper [53]. Additionally, 30 larvae per family were preserved in RNAlater (Sigma-Aldrich, Germany) at each sampling point for transcriptomic (at MO) and qPCR (at MO, weaning and at the end of larval period) analysis.

### RNA extraction

Total RNA was extracted from larvae at three different time points (Fig. 1b; MO stage, weaning and at the end of larval period) using a TotalRNA mini-kit (A&A Biotechnology, Poland). Specifically, for each family, RNA was extracted from pool of 10 larvae at MO stage (mean WBW 10.0 ± 0.5 mg). For larvae at the weaning, RNA was isolated from pool of four larvae per family (mean WBW 76.6 ± 1.8 mg), and for larvae at the end of larval period, from pool of three larvae per family (mean WBW 180.3 ± 5.6 mg). After extraction the concentration and purity of the RNA were assessed with a DS-11 spectrophotometer (DeNovix, USA), showing absorbance ratios of A260/280 ≥ 2.0 and A260/230 ≥ 2.2. RNA quality was further confirmed using the Agilent Bioanalyzer 2100 (Agilent Technologies, USA), with all samples exhibiting RIN values ≥ 9.0. Importantly, only RNA samples from larvae at the MO stage were then sent for transcriptomic analysis. RNA extracted from MO and other sampling points were used for Real-time qPCR validation.

### RNA sequencing

Twelve different libraries were created from larvae at the MO stage (six families for CON and six families for PTS). RNA-seq analysis was outsourced to Macrogen (Amsterdam, Netherlands) using the TruSeq Stranded mRNA kit (Illumina) with a NovaSeq6000 platform, and 40 M 150 bp paired-end reads per sample were generated.

### Bioinformatic analysis

Raw reads were subjected to quality control using FastQC software version 0.11.9 [54]. Adapters and low-quality fragments of raw reads (average *QPhred* score < 20) were trimmed, and reads were trimmed to equal lengths of 140nts using Trimmomatic ver. 0.40 [55]. The resulting sets of reads of the analyzed samples were mapped to the *P. fluviatilis* reference genome version 11.1.104 obtained from the NCBI database [56] using STAR software version 2.7.10a [57] with default ENCODE options. Transcript count data for larval samples were filtered to include at least five libraries with at least five reads. Libraries with 0 min and 30 min PTS were compared using the following scheme: ~ *families + condition*; families representing the 6 males observed during the experiment and condition representing 0 min and 30 min PTS. Differential expression analysis was performed in RStudio (version 4.1.3) using the DESeq2 package and *ashr* normalizing the fold change value by logarithm [58]. Results were filtered with thresholds absolute value of log2FC > 1 and q-value < 0.05; differences were considered as significant when adjusted p-values were less than ɑ (ɑ = 0.05).

The transcriptomic expression data (in transcripts per million; TPMs) were first fed to DataMap (version 0.11; [59]), which identified the 100 most variable genes. The dataset was subsequently normalized by z-score transformation, standardizing each row to a mean of 0 and a standard deviation of 1. A heatmap of hierarchically clustered genes was generated using “row clustering” across biological replicates (Families A–F) under the CON vs. PTS groups. Thereafter, the differentially expressed genes (DEGs) were similarly transformed and subjected to two heatmap analyses: one utilizing “supervised clustering” and the other “unsupervised clustering.” Unsupervised clustering was based on column clustering, internally applying distance metrics and linkage algorithms to group the columns using DataMap. In contrast, in the supervised approach, manual clustering was applied to organize samples into two groups, ensuring that each male under CON and PTS conditions remained paired. A Principal Component Analysis (PCA) plot [60] using iDEP, and clustering of genes using GeneMANIA/ Cytoscape [61, 62]) were generated for visualization.

### GOEA analysis

Gene Ontology Enrichment Analysis (GOEA) has been employed to elucidate the biological significance of DEGs. For assessment of gene functions, we used ShinyGO platform [63], preceded by BLAST search of Eurasian perch transcriptome obtained against the human Swiss-Prot protein dataset. Only the top match for each protein was taken, which provided gene names and UniProt accession numbers for the aligned proteins. In the absence of enriched biological pathways among the DEGs using the ShinyGO platform, we extracted the gene table that was grouped by functional categories that were defined by high-level GO terms (Additional file, sheet 2). Furthermore, GeneMANIA platform [61] was utilized to infer putative gene functions and to construct interaction networks by integrating data from publicly available databases. Among the 31 identified genes, *mfap4* appeared in four instances, each localized to distinct chromosomal regions, while *gimap* was represented by two orthologs, *gimap4* and *gimap7*. GeneMANIA successfully networked 27 genes that network with physical interactions, available co-expression datasets, and shared protein domains. The interactions were further visualized using Cytoscape.

### qPCR validation

Among DEGs, genes chosen for validation based on their consistency in expression patterns, meaning all biological replicates following similar trends of their relative expressions (either upregulated or downregulated). Next, the selected genes were checked for their maternal origin, meaning whether they were expressed in unfertilized eggs (UFE) (based on our previously obtained transcriptome of UFE, with threshold of expression >1TPM; ([27] Additional file, sheet 3), and as PEAGs only genes not expressed in UFE genes were considered. Primers were designed for such genes (Table 1), and once they were found to be positively validated using RT qPCR at MO stage, the expression of those genes was also checked for larvae at weaning and at end of larval period.

**Table 1:** Primers designed for the chosen genes for validation with qPCR, along with normalizing genes

	Genes	Accession number	Reference	Forward (5’-3’)	Reverse (3’-5’)	Size (bp)
Normalizing genes	*kdm1a*	XM_039786859.1	NC_053131.1	GAAGCAGGTCATCCCTCCAC	GCCTCCTGGGATGTCATACG	132
	*stmn1b*	XM_039786859.1	NC_053118.1	AAACTGGAAGCGGCAGAAGA	TTGAGAGCTGCCATCCTTGC	203
	*coq9*	XM_039809994.1	NC_053119.1	GGCTTCCAGGCTAAACACCA	CCAGTGTCTCAGCACCGATT	164
	*atp6v1ba*	XM_039783937.1	NC_053130.1	TTTGCCATTGTCTTCGCAGC	GTCATTGGCCAGGTTGAGGA	120
	*taf13*	XM_039799669.1	NC_053116.1	GGAGCTCCGGTGTATGATGT	CTTTGTGGGTCATTTCCGTGA	113
Differentially expressed genes	*sxt(chr13)*	XM_039820350.1	NC_053124.1	TCCTGGTCTGGTCTGTCACT	TGTGACCATGACCGTCTTGT	113
	*pde6g*	XM_039820828.1	NC_053112.1	AAGAAGGCCCCTCCCAGATT	GCAGAGGGAAGAGGGCTAAA	210
	*trim16*	XM_039787215.1	NC_053132.1	CCCAGGTTTGACATTGTGGG	CAGGACACTGGCCTTCAGAG	154
	*alox15b*	XM_039822191.1	NC_053125.1	AGGACTATCGCTGGCATGTG	CAGTGGCTGCAGTGAAGAGA	132
	*plaat4*	XM_039787023.1	NC_053131.1	GGAAAGTGGTCGGCAATGATA	AGTGCTCGCAGTTGCTATTCAC	150
	*ralgapb*	XM_039798363.1	NC_053115.1	ACGGCTTGACTCTCCCATTG	GGTTGGGCTCTCTGCTGATT	126
	*neo1*	XM_039804040.1	NC_053117.1	CAGAGCACCCACTGGTTTCT	TCACGTGTCTGATGCAGCAT	127
	*gimap4*	XM_039783037.1	NC_053130.1	TCCTTGTAGGCCTAGTGTCTGA	TGTTGGAGCCATATGGACCA	113
	*mfap4(chr10)*	XM_039813507.1	NC_053121.1	GCAGGAAAGTAGGGCCCATA	ACTGGATGTCCTGAACTGCA	164
	*mfap4(chr11)*	XM_039815040.1	NC_053122.1	CCTGCTCGGTCTCTGTTTCA	TGACACGCCAGCACTTTTTG	135
	*mfap4(chr18)*	XM_039781790.1	NC_053129.1	CGGTAGACTGCGGTGACATT	CACAGTACACCTGGACAGCA	102
	*mfap4(chr21)*	XM_039788849.1	NC_053132.1	TCAGGCACCGTTTAGGAACC	TTTTCTTCCACGTTGCAGCG	144
	*fads2*	XM_039808830.1	NC_053119.1	ACAGTACCTACAAGGACTGCTG	GTGACGTCCCTGACACCATAC	216
	*pigr*	XM_039821933.1	NC_053125.1	AGCTCTGAGTTCCTCTAGGGT	TCTCTGATGCAACTACTACTCG	132
	*dmbt*	XM_039781339.1	NC_053129.1	CTGGCCCAATATGGCTGGAT	ATGAGCCAGGCCTCTTACCTA	144
	*hlag*	XM_039820208.1	NC_053124.1	GGCTGCATCATCCTCTGGTTA	TCTGTGTGCAGGATGGGTTTA	134

Primers for 16 PEAGs chosen for validation, along with five normalizing genes for RT qPCR, were designed using Primer3Plus software version 3.3.0 [64]. The primers were confirmed for their specificity using NCBI-PrimerBLAST, version 1.0.1 [65]. The predicted amplicon sequence was then checked for melting temperature (Tm) on µMelt Quartz, version 3.6.2 [66]. The normalizing genes were chosen according to their lowest coefficient of variation (CV = standard deviation/ mean expression *100) within the transcriptomic data obtained [67]. The best matching families were chosen based on their least possibilities to form secondary structures and GC content. For the four *mfap4* genes chosen for validation, we used Clustal Omega [68], a platform to align multiple sequences, and primers were designed on the most unique regions possible (Additional file, sheet 4). For easier understanding of the gene paralogs, they have been named differently, with their chromosome numbers (chr) provided along with their names. The primer’s specificity was then verified using the NCBI Primer-BLAST tool.

RT‒qPCRs were performed for each gene using a Viia7 (Applied Biosystems) thermocycler. For each qPCR, 10ng cDNA template was used along with 10 µl SYBR RT PCR Master Mix (A&A Biotechnology), 0.5 µM forward (1 µl) and reverse (1 µl) primers, 2 µL of starter mix and PCR grade water were added to maintain a final volume of 20 µL. The reactions were performed with the following cycling conditions applied: enzyme activation for 10 min at 95 °C, followed by 40 cycles of denaturation at 95 °C for 15 s and annealing and elongation at 60 °C for 1 min. For each gene, first, a standard curve was performed using serial dilutions (six 2-fold dilutions) of the mixture of cDNA templates to verify the reaction efficiency. Reaction specificity has been assessed with the melt-curve analysis and compared to µMelt prediction. Next, the quantification of expression has been performed for each gene and for each sample separately, which was then followed by analysis of their relative expression using the delta delta Ct (2–ΔΔCt) method [69]. Relative expression for each gene was normalized as the geometric mean of expression values recorded for 5 normalizing genes (namely, lysine demethylase, *kdm1a*; stathmin 1b, *stmn1b*; ubiquinone biosynthesis, *coq9*; ATPase H+ Transporting V1 Subunit B2, *atp6v1ba*; and TATA-Box Binding Protein Associated Factor 13, *taf13*).

### Data analysis and statistics

All the data passed Shapiro Wilk test for normal distribution, and thus they were proceeded to conduct parametric tests. The obtained data from sperm motility parameters (in %, µm s^–1^), were analyzed using one-way ANOVA between fresh, CON and PTS groups. Fertilization and embryonic developmental rates (%), deformities (%), SBIE (%), TL (mm), and WBW (mg) were analyzed using paired t-tests between CON and PTS groups. Other parameters like cumulative mortality and cannibalism rates (%) during larviculture were subjected to 2-way ANOVA. All analyses were performed at a significance level of *p* < 0.05 using GraphPad Prism Software (USA). Similarly, relative expression values after RT-qPCRs were first analyzed with paired t-tests with GraphPad and later plotted using the Matplotlib package [70] in Python.

## Results

### Cryopreservation and PTS impair sperm motility parameters

The milt quality was ensured using sperm motility parameters, sperm concentration (23.7 ± 3 × 10^9^ spermatozoa/mL) and sperm viability (84.2 ± 3.4%). Cryopreservation decreased all sperm motility parameters, except linearity (Fig. 2a-e), compared with fresh milt. Furthermore, storing milt at 4 °C for 30 min reduced all CASA parameters compared with both fresh and control (CON) milt.

### PTS increases larval mortality

**Fig. 3:**
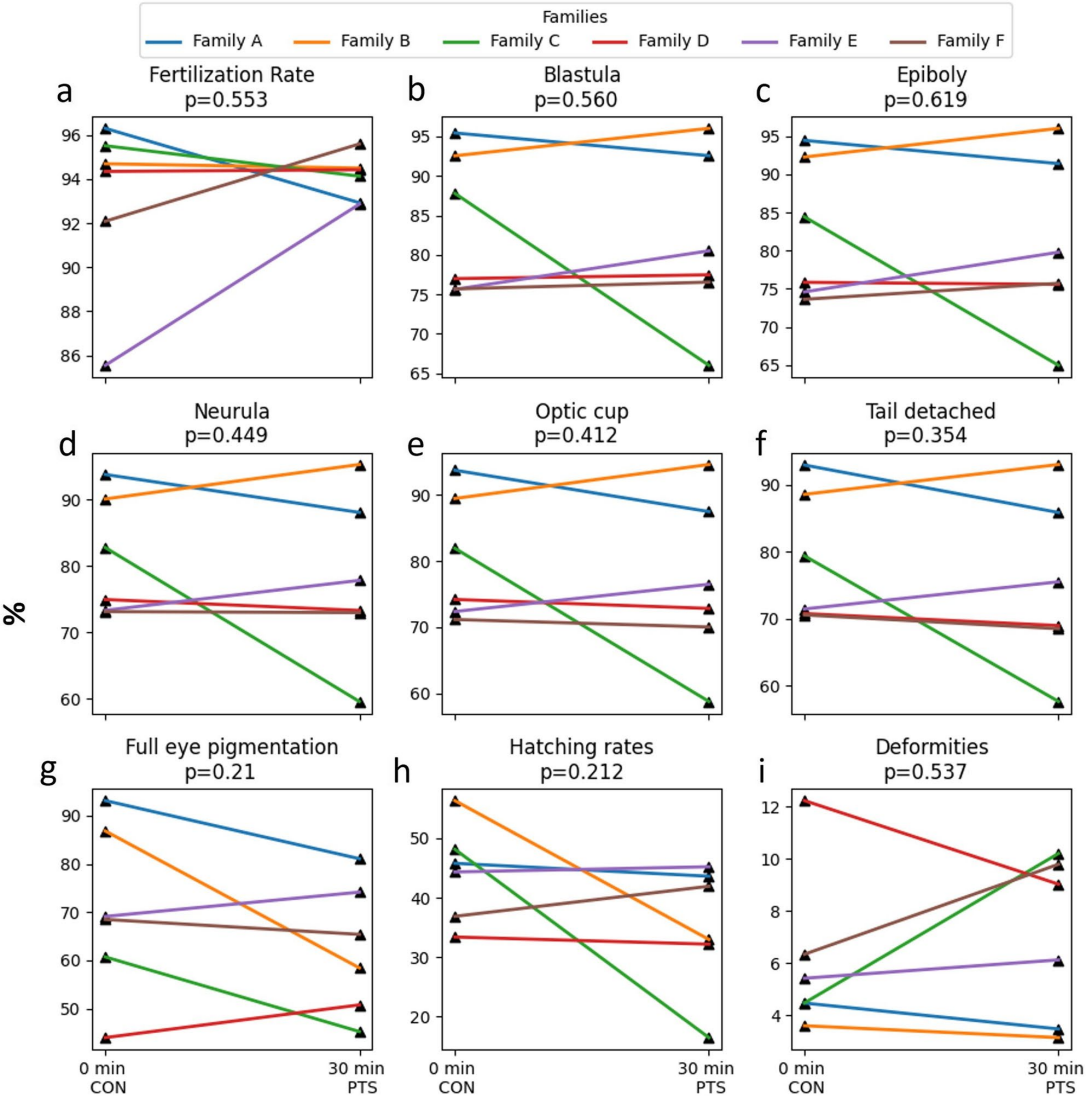
Early life history traits. **a** Fertilization; (**b**-**g**) embryonic developmental; (**h**) hatching and (**i**) hatched larvae deformity rates recorded in Eurasian perch embryos and larvae obtained with cryopreserved sperm either 0 min post-thaw (control group; CON) or with 30 min post thaw-stored (PTS group) sperm. No statistical differences were observed

**Fig. 4:**
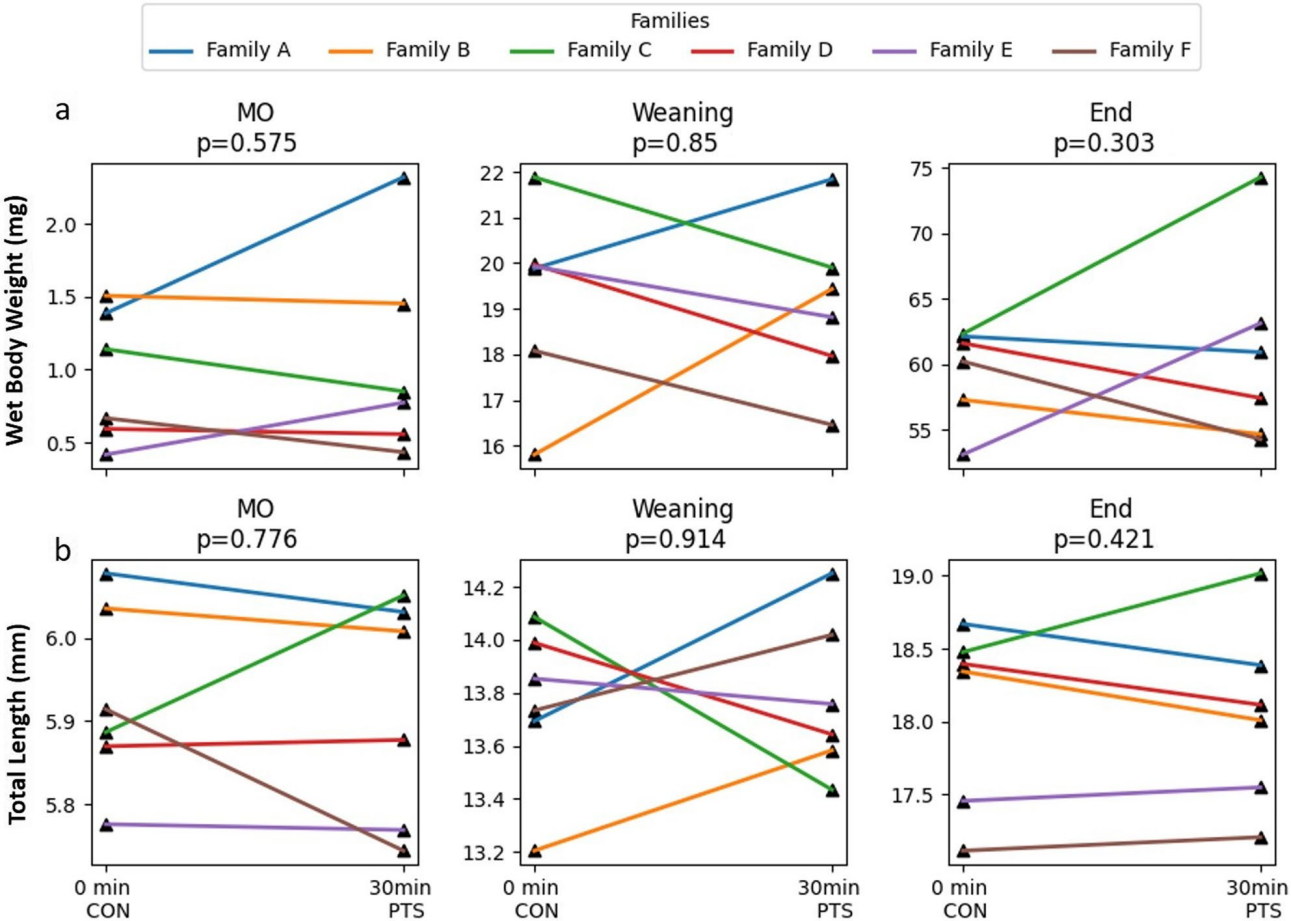
Zootechnical parameters - Growth indicators. Wet body weights (mg) and total lengths (mm) of larvae at mouth opening (MO) stage, weaning and end of larval period. 0 min post-thaw (control group; CON); 30 min post thaw-stored (PTS group) sperm. Lack of superscripts indicate no significant differences among the groups

**Fig. 5:**
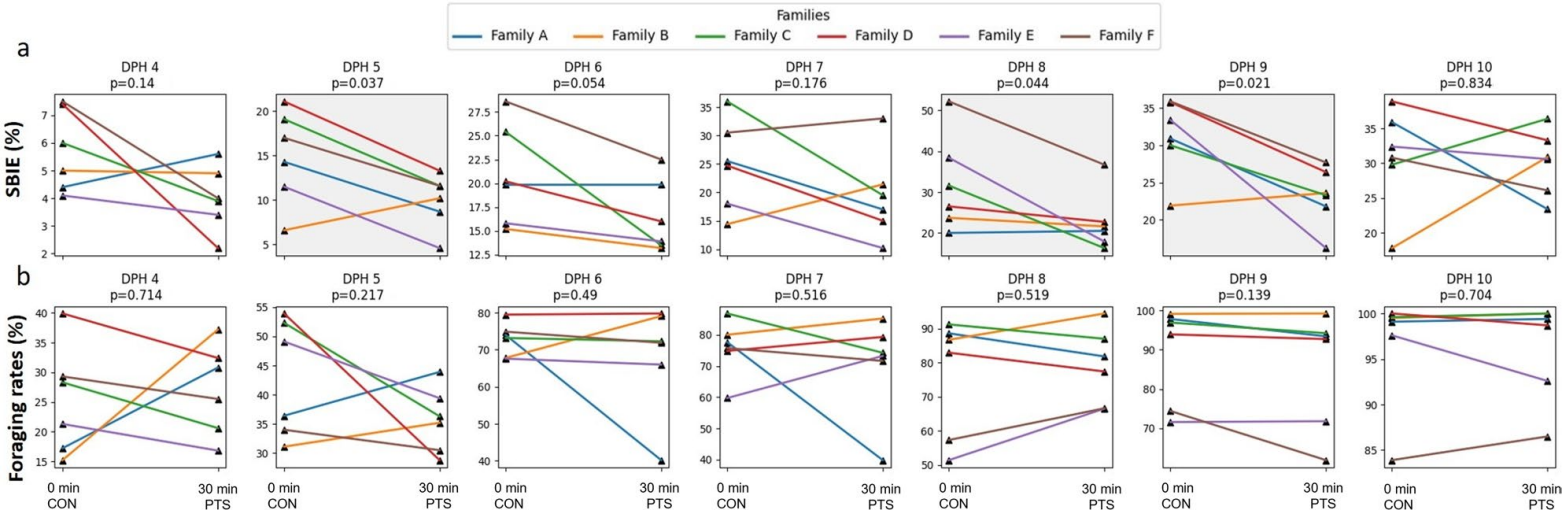
Zootechnical parameters – Functional performance. **a** Swim bladder inflation effectiveness (SBIE, %) measured during early larval development. Data in bounding boxes in grey for certain days with their *p* values (< 0.05) highlighted were significantly different. **b** Foraging rates (%) of larvae from the first day of exogenous feeding (4 DPH) until 10 DPH. 0 min post-thaw (control group; CON); 30 min post thaw-stored (PTS group) sperm

**Fig. 6:**
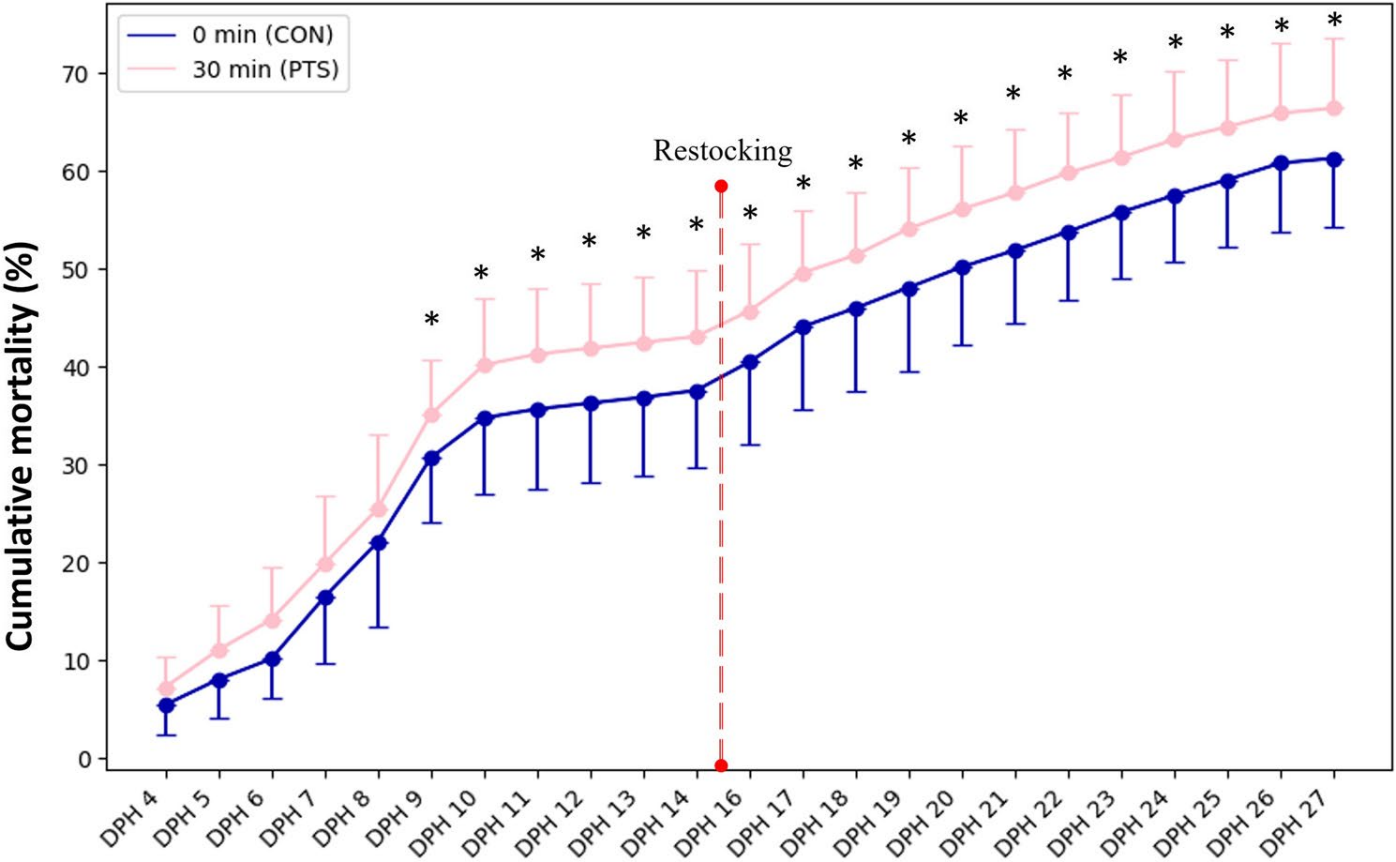
Cumulative mortality (%) recorded along the larvicultural period. On day 15 larvae were restocked to equalize the number of larvae in each tank before weaning them into commercial compound feed. Data marked with an asterisk for each day indicates statistical difference between CON and PTS (*p* < 0.05). 0 min post-thaw (control group; CON); 30 min post thaw-stored (PTS group) sperm

**Fig. 7:**
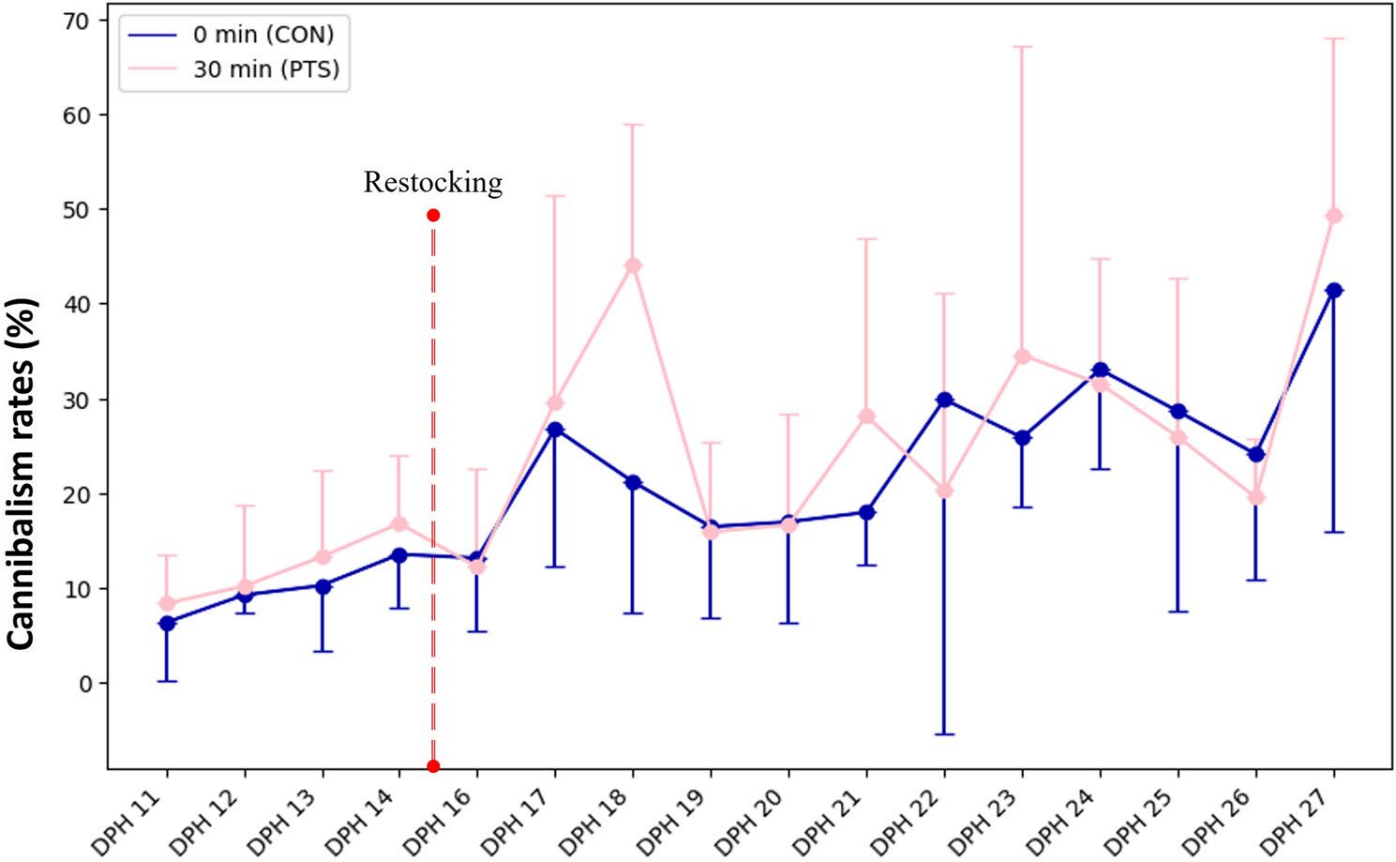
Type I cannibalism rates (%) of larvae recorded during the larvicultural period. On day 15 larvae were restocked to equalize the number of larvae in each tank before weaning them into commercial compound feed. Data between the groups for each day of rearing were not statistically different (*p* > 0.05). 0 min post-thaw (control group; CON); 30 min post thaw-stored (PTS group) sperm

No significant differences (*p* > 0.05) were found in the fertilization rates (Fig. 3a), embryo developmental stages (Fig. 3b-g), hatching rates (Fig. 3h), and larval deformity between CON and PTS groups (Fig. 3i). Lack of significant differences were also noted in TL and WBW throughout the larviculture period (Fig. 4a-b). There were significant differences (*p* < 0.05) in SBIE between CON and PTS groups on 5 DPH, 8 DPH, and 9 DPH (Fig. 5a), unlike foraging rates between families in CON or PTS conditions (Fig. 5b). Mortality rates in the PTS group were statistically higher (*p* < 0.05) compared to the CON group, starting from oil droplet reduction (9 DPH) until the end of the larval period (Fig. 6). There was no significant difference (*p* > 0.05) in the case of cannibalism between the CON and PTS groups during larviculture (Fig. 7).

### Transcriptomic profiling

**Fig. 8:**
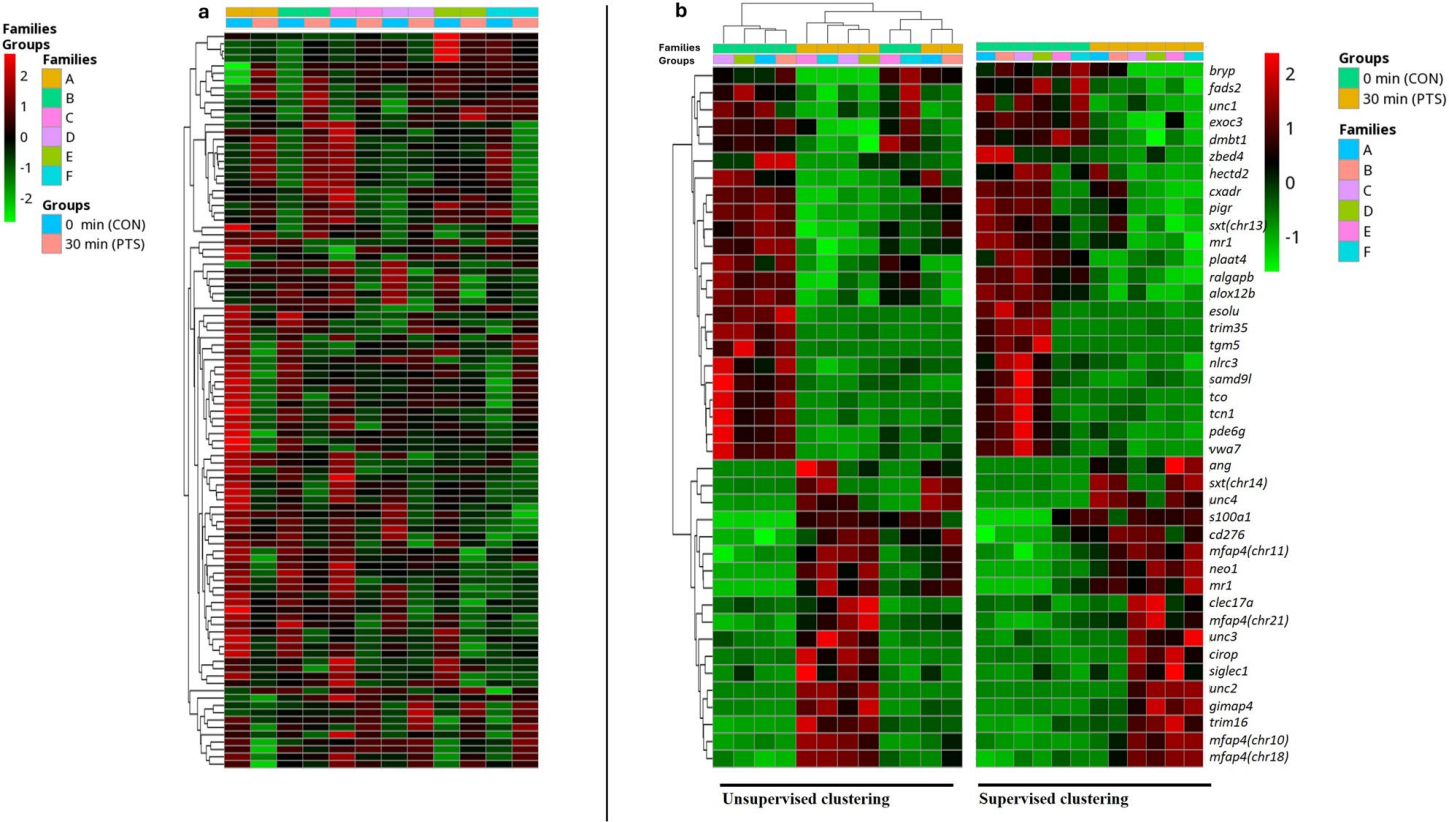
Transcriptomic profiling of Eurasian perch larvae at MO. **a** Heatmap showing hierarchical clustering of 100 most variable genes in the transcriptome of Eurasian perch larvae at MO (red and green bricks represent over- and under-expression levels, respectively while black color represents median variability of the gene). **b** Heatmaps showing unsupervised and supervised clustering of the obtained 41 differentially expressed genes (DEGs; red and green bricks represent over- and under-expression levels, respectively while black color represents median variability of the gene). MO – Mouth opening; 0 min post-thaw (control group; CON); 30 min post thaw-stored (PTS group) sperm

**Fig. 9:**
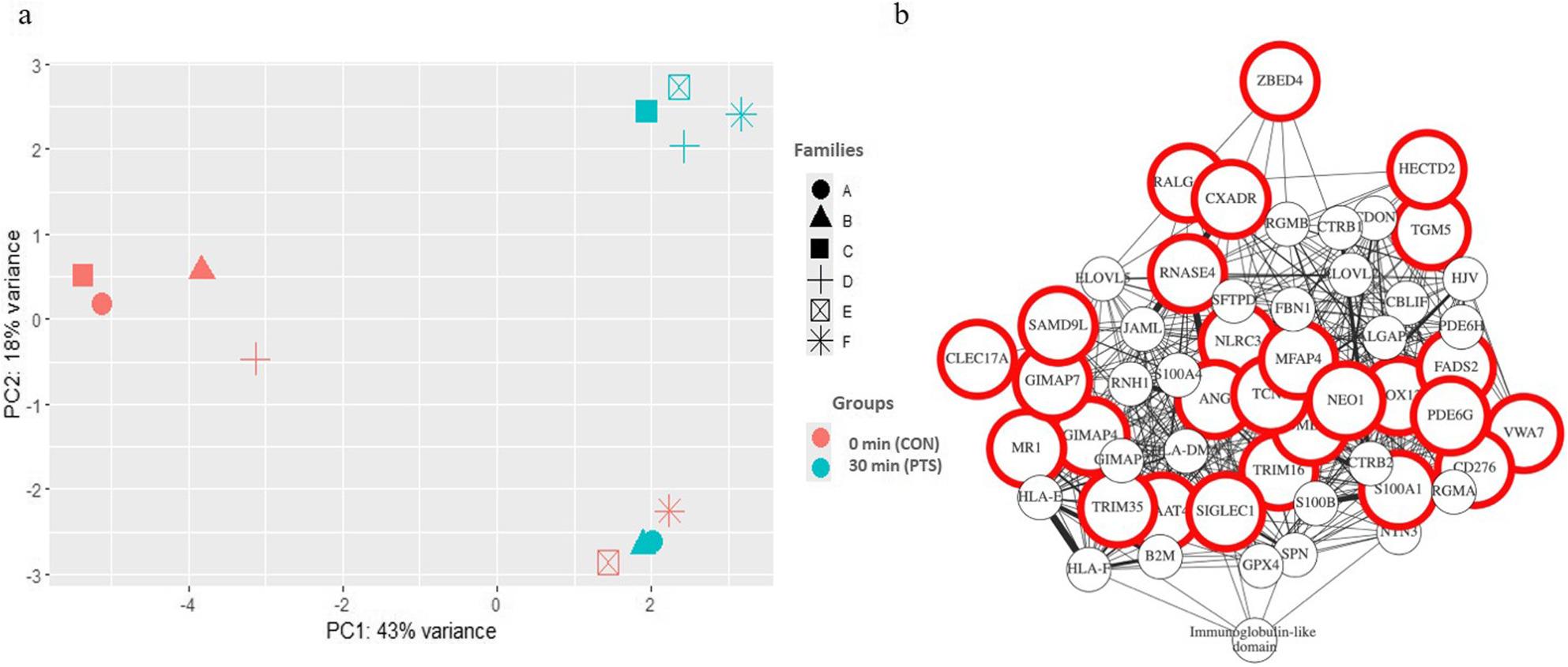
Multivariate and network analysis for transcriptional signatures in CON and PTS groups. **a** Principal component analysis of CON: 0 min and PTS: 30 min group showing the dispersion of families created, represented using symbols, while colors represent the groups (CON and PTS). **b** Genes clustered according to their co-expressions, shared protein domains and physical interactions with other genes. The gene names encircled in red are our differentially expressed genes. 0 min post-thaw (control group; CON); 30 min post thaw-stored (PTS group) sperm

A transcriptomic profiling of larvae at mouth opening was conducted to identify DEGs between CON and PTS groups. Following RNA-seq analysis, a total of 30,744 genes were initially identified. After applying filtering criteria for expression level, 20,447 unique protein-coding genes remained, which were then used for the differential analysis to identify potential PEAGs. Heatmap showing hierarchical clustering of 100 most variable genes in the transcriptome of freshly hatched larvae clearly showed differences between CON and PTS (Fig. 8a). The heatmaps show three distinct clusters formed when not supervised, also indicating a maternal driven pattern (Fig. 8b). PCA of transcriptomic data revealed a total explained variance of 61%, with PC1 accounting for 43% and PC2 for 18% (Fig. 9a). In most male-female combinations (families A–D), samples from the CON and PTS groups showed clear separation along PC1. In contrast, families E and F exhibited limited separation along PC1 but greater dispersion along PC2. The genetic background within each pair was constant, and treatment conditions were identical, yet variation in transcriptomic profiles differed in both direction and magnitude across families. The families are seen to be driven in a female-driven manner as the families seem to vary based on their paired female.

Gene ontology analysis revealed lack of enrichment in any biological processes, however, they did group into some high-level Gene Ontology categories (see Additional file, sheet 3). Out of 41 DEGs, for 31 genes human protein ortholog has been found. The highest number of genes (*n* = 9) is responsible for ‘regulation of response to stimulus’, followed by ‘immune response’ processes (*n* = 8). Upon checking physical interactions among DEGs (Fig. 9b) the genes seem to form a dense network connecting *mr1*, *gimap*, *plaat*, *samd9l* and *siglec1*, along with their orthologs in humans, among many other such clusters. The genes clustering around our DEGs in the GeneMANIA/ Cytoscape network likely represent functionally associated genes that may contribute to or modulate the biological processes regulated by the DEGs.

**Fig. 10:**
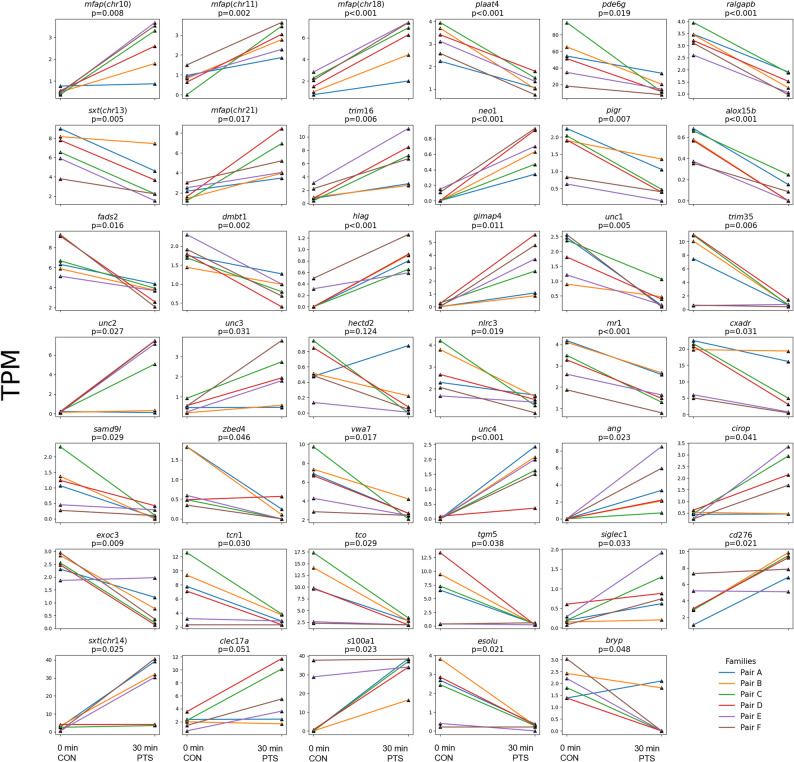
Graphs showing gene expression changes (up/downregulated) relative to CON group in normalized TPM values. For mfap4 genes in the parentheses the gene name has been supplemented with information on number of chromosomes it originates from. 0 min post-thaw (control group; CON); 30 min post thaw-stored (PTS group) sperm; unc – uncharacterized gene; chr - chromosome; TPM – Transcripts per million

**Fig. 11:**
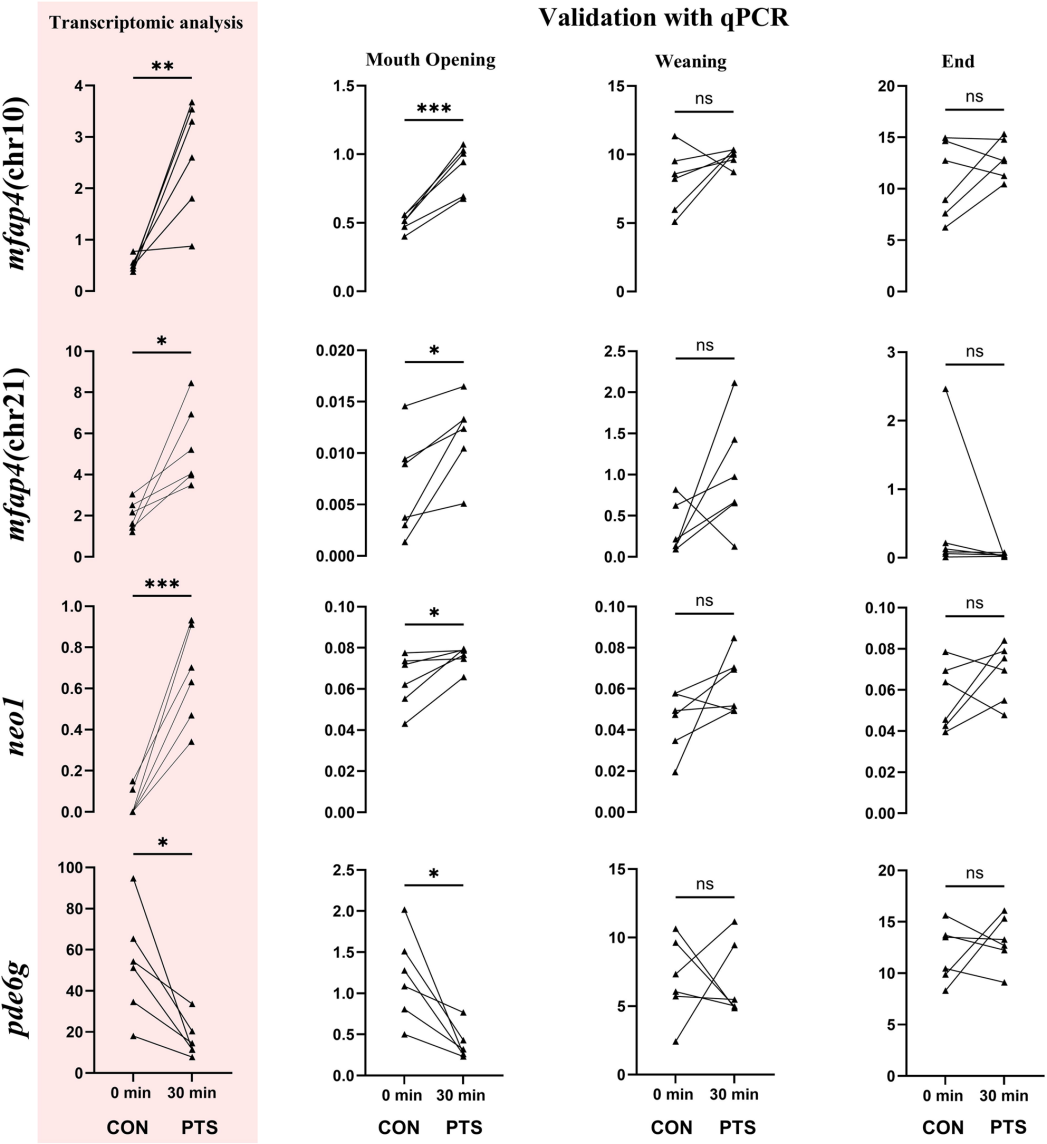
Graphs in pink panel represent the candidate paternal-effect-associated genes chosen for validation after transcriptomic analysis. Validation using RT-qPCR was done for RNA from larvae at mouth opening, weaning, and end of larval period. Significant differences in expression among the groups was seen only at the MO stage. (*p* < 0.05 = *, *p* < 0.01 = **, *p* < 0.001= ***). 0 min post-thaw (control group; CON); 30 min post thaw-stored (PTS group) sperm

**Fig. 12:**
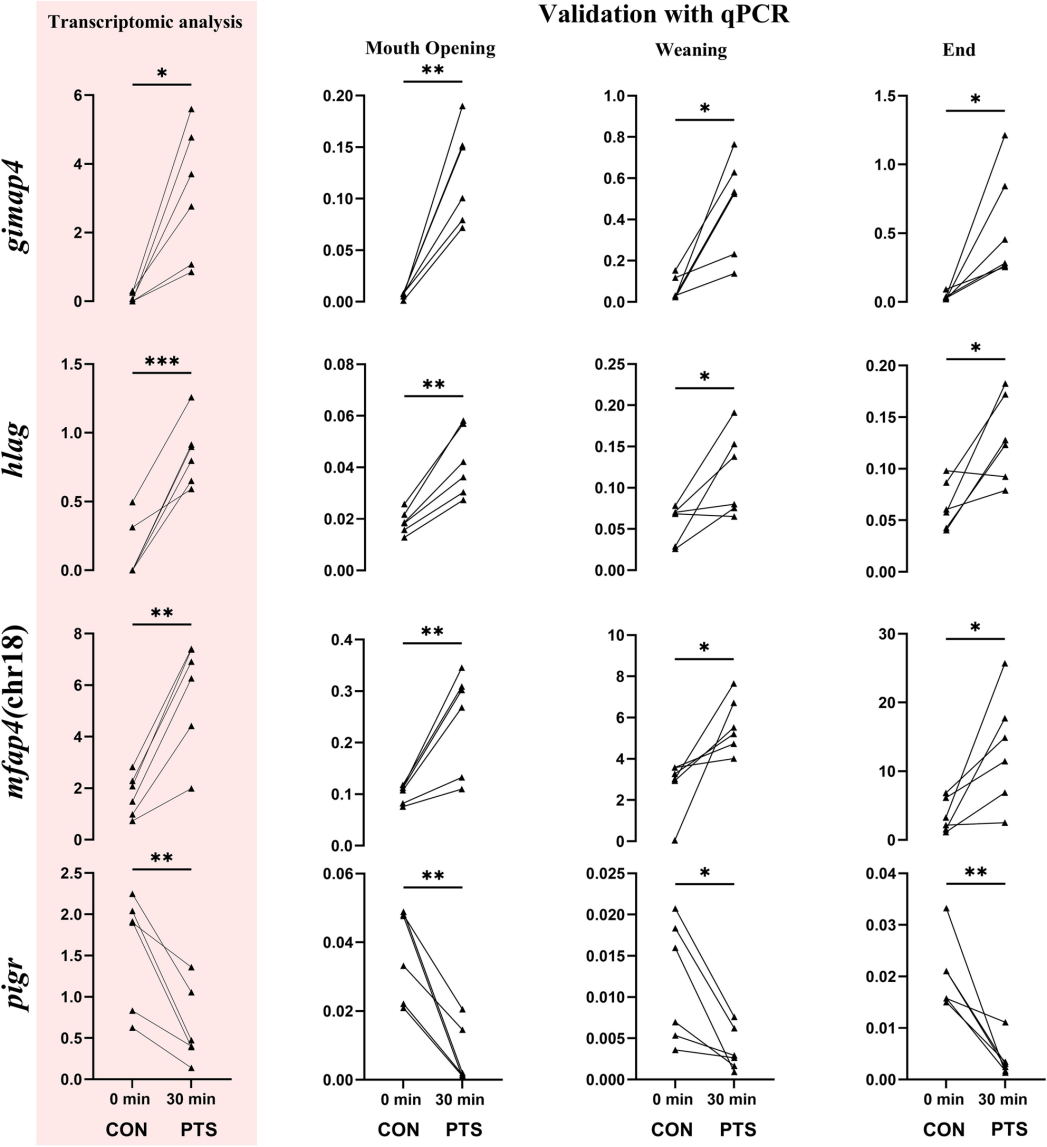
Graphs in pink panel represent the candidate paternal-effect-associated genes chosen for validation after transcriptomic analysis. Validation using RT-qPCR was done for RNA from larvae at mouth opening, weaning, and end of larval period. Significant differences in expression among the groups was seen at all three considered stages. (*p* < 0.05 = *, *p* < 0.01 = **, *p* < 0.001= ***). 0 min post-thaw (control group; CON); 30 min post thaw-stored (PTS group) sperm

Of the 41 DEGs, 16 genes were selected for RT-qPCR validation (Fig. 10), of which 8 were positively validated at the MO stage (Figs. 11 and 12). Genes that were upregulated in the PTS group and positively validated only at MO stage include *mfap4* (chr21), *mfap4* (chr10), and *neo1*, on contrary to downregulated in the PTS group is *pde6g* (Fig. 11). The expression level of these was similar in both groups at weaning and end of the larval period (27 DPH) suggesting their expression compensation along the larval metamorphosis. The genes that maintained differential expression also at weaning, and 27 DPH include three upregulated genes in the PTS group [*mfap4*(chr18), *gimap4* and *hlag*], and one downregulated gene in the PTS group - *pigr* (Fig. 12). The genes selected for validation but not confirmed at the mouth opening stage are presented in Additional file, sheet 5.

The microfibril-associated glycoprotein 4 (*mfap4*) gene is present on five chromosomes in Eurasian perch (chr10, chr11, chr18, chr19 and chr21) and is therefore considered to consist of paralogs occupying distinct loci, which may evolve new or specialized functions over time. Out of those five different loci, the gene transcripts from four chromosomes have turned out to be PEAGs. Furthermore, two of them, *mfap4*(chr21), and *mfap4*(chr10), were validated positively and have significant differences until the MO stage, and third one, *mfap4*(chr18), is not just positively validated but also is seen to maintain significantly different expression along the larval period. Importantly, the three (validated) differentially expressed paralogs of *mfap4* have higher expression in the PTS group, showcasing the strong candidature of the gene as PEAGs.

## Discussion

In this study, we show that even short-term post-thaw storage of cryopreserved sperm can induce significant changes in both gene expression and phenotype in Eurasian perch offspring. Using a controlled, paired fertilization design, sperm first underwent selection for robustness and further potential accumulation of sub-lethal damage during storage. We observed a consistent decline, rather than an improvement, in progeny performance in experimental groups. However, we cannot determine whether the observed differences arose from environmentally induced modifications of the sperm molecular cargo, from additional selection favoring the most robust sperm surviving PTS, or from a combination of both mechanisms. Nevertheless, transcriptomic responses to PTS across progenies from different families helped identify several novel candidate paternal-effect-associated genes (PEAGs). These findings suggest that PTS imposes selective pressure on sperm, thereby differentially programming the resulting offspring. Interestingly, the magnitude and pattern of these responses appeared to be female-dependent, indicating that maternal identity may modulate the impact of PTS on non-genetic inheritance carried by sperm.

There is currently limited research specifically examining the effects of PTS on larvae characteristics. The majority of existing work evaluates sperm quality metrics, particularly motility, within minutes to hours after thawing [71, 72], while fewer studies examine fertilization success, and almost none extend assessments to later developmental stages. In this context, our study presents novel findings, demonstrating that larvae derived from post-thaw stored milt in Eurasian perch exhibit significantly increased mortality beginning at 9 days post-hatching. This suggests that sub-lethal cryo-induced damage, may have delayed effects on embryonic or larval survival. We also documented a progressive decline in sperm quality, as reflected by motility parameters, from fresh milt to 0 min post-thaw and further to 30 min post-thaw. These observations are consistent with previous findings [38, 73] who reported that even under optimized cryopreservation conditions, perch sperm undergoes a rapid decline in motility within the first few minutes post-thaw, reaching approximately 54% after 30 min. Such early post-thaw deterioration is atypical among fish, and to our knowledge, no comparable rapid decline has been reported in other species. While the exact mechanisms remain to be fully elucidated, we speculate that this phenomenon may be related to spontaneous activation of perch spermatozoa in seminal plasma, a phenomenon we previously observed in E. perch [38]. Such spontaneous motility has also been observed in other percid species, pikeperch (*Sander lucioperca*; [74]). This phenomenon may possibly be responsible for early energy depletion or cellular stress, thereby exacerbating the decline in motility shortly after thawing. On the other hand, in salmonids such as rainbow trout (*Oncorhynchus mykiss*), brown trout (*Salmo trutta*), or Adriatic grayling (*Thymallus thymallus*), post-thaw sperm can maintain high fertilizing ability for up to 1 h, with gradual declines observed thereafter [75]. Even in species such as African catfish (*Clarias gariepinus*), sperm stored post-thaw for 24 h can retain fertilization capacity, although rates drop markedly by 96 h [76]. These differences emphasize that PTS tolerance is highly species-specific and affected by sperm physiology, cryoprotectants, and extender composition [77]. Despite the physiological deterioration observed, cryopreserved sperm has been successfully used in controlled fertilization in various species for research or genetic conservation [35, 36, 78–80]. However, our findings go a step further, demonstrating for the first time that short-term PTS can affect larval survival, providing compelling evidence that conventional quality assessments (e.g., motility, fertilization rate) are not sufficient to ensure progeny viability. Importantly, this short post-thaw window of sperm functionality in perch, which has often been viewed as a limitation, can be repurposed as an experimental tool. The predictable and rapid decline in milt quality offers a unique opportunity to experimentally manipulate sperm condition, enabling researchers to investigate the role of paternal-effect-associated genes and non-genetic paternal contributions to early development. To our knowledge, this is the first study to link PTS with altered larval phenotype, representing a significant step forward in understanding the long-overlooked role of paternal quality in fish development.

We have demonstrated that many parameters of the early life stages of larvae, like hatching, deformity, feeding rates, etc., do not seem to be affected significantly by the PTS of milt compared to CON group. Though significant differences in SBIE which is a crucial zootechnical parameter [81], there is no strict pattern being followed to claim this parameter to be paternally driven. Similarly, we did not observe any differences in Type I cannibalism (sibling cannibalism) between our comparison groups, despite this behavior being a common trait in the Percidae family [50]. This trait is typically influenced by ecological conditions rather than parental control, particularly under captive conditions. In our previous study, we speculated that foraging effectiveness has been modified by the cryo-selection of the sperm and attributed to the modified visual system as paternally-controlled trait [27]. However, in this study we did not see the differences in foraging until 10 DPH, what suggests that higher mortality after the yolk sac depletion can stem from impaired digestion capacity of exogenous food or metabolism [82]. Thus, larval mortality can stand alone to be a very strong result from this experiment, after all, mortality is a powerful phenotype to assess larval quality [83].

The overview on transcriptomic data demonstrates that PTS introduces consistent, condition-driven gene expression changes in a subset of larval transcriptomes, supporting the use of PTS as a stressor for identifying paternal contributions to early gene regulation in the progeny. For instance, the visualization of 100 most variable genes (Fig. 8a) already shows clear partitioning between most CON and PTS samples, suggesting a strong effect of PTS on global gene expression. When focusing on the 41 DEGs the supervised clustering maintains the female-based family structure and reveals that, in several families, PTS samples display consistent shifts in expression relative to their CON counterparts. This indicates that, despite shared genetics, sperm exposed to PTS undergo changes that alter larval transcriptional profiles [84]. In contrast, the unsupervised clustering organizes samples based solely on expression similarity, and here, we observe that CON and PTS samples often group separately, highlighting that PTS introduces a reproducible transcriptomic signature [85]. However, some variability remains, particularly in families E and F, where the distinction between treatments is less pronounced. This suggests that not all males’ sperm respond equally to post-thaw storage stress, hinting at male-specific resilience [85, 86]. Further analysis of transcriptomic data revealed substantial intergroup variability, and low number of DEGs observed. PCA analysis showed that most families (A–D) exhibited a shared, time-dependent transcriptional response to PTS, with clear separation along PC1. In contrast, families E and F showed minimal separation along PC1 but greater dispersion along PC2, indicating a distinct source of variation. Since the genetic background was held constant within each pair, this divergence could reflect individual differences in how sperm from these males responded to post-thaw storage stress (Fig. 9a). Notably, families sharing the same female (A–B, C–D, E–F) dispersed similarly along the principal components, suggesting a potential maternal effect modulating the offspring’s response to PTS-altered sperm [87, 88]. This variability may arise from interactions between sperm-borne non-genetic changes, such as epigenetic modifications or small RNA profiles, and female-specific oocyte environments [89, 90]. Although the exact mechanisms are still unclear, these findings suggest that both paternal tolerance to PTS stress and maternal factors work together to influence early gene expression in the offspring. This highlights complex, context-dependent parental effects observed under controlled experimental conditions.

The transcriptomic profiling of CON and PTS groups of larvae at the MO stage is essential as the larvae would have been least manipulated by human intervention. Results indicate that the female may be an important component influencing how paternal effects are expressed. While the sperm’s main contribution to the embryo is its compacted DNA, it also delivers, for example, small RNAs and epigenetic marks that can influence early development [1]. However, when sperm DNA is damaged, for example due to oxidative stress, membrane instability, or fragmentation during PTS [91], the embryo must rely on internal repair systems to maintain developmental potential. In fish and other vertebrates, these repair mechanisms come mainly from the oocyte cytoplasm and play a central role in recognizing and fixing such damage, especially before or during zygotic genome activation [92]. For instance, in rainbow trout, when the base excision repair (BER) pathway was inhibited using a PARP inhibitor (3AB), it caused increased embryonic mortality and genome instability [93]. Similarly, studies in zebrafish showed that moderate levels of DNA damage in sperm can be tolerated if the maternal repair machinery is functioning properly [94]. However, these repair systems are not completely effective; excessive or unrepaired damage can result in apoptosis or developmental arrest. Also, while DNA can often be repaired, epigenetic changes caused by cryopreservation, such as abnormal methylation or histone modifications, may not be fully corrected and could still be passed to the offspring [91, 92]. So, although the egg can compensate for some damage, this alone does not explain the full variation we observed in transcriptomic profile. Despite the mechanism of such maternally-shaped variability in paternal contribution to offspring transcriptomic repertoire is unclear, our results suggest that the paternal effect is shaped not only by sperm-derived factors, but also by how the egg responds to it indicating a complex interaction between maternal and paternal factors in shaping the larval phenotype.

Despite the variability in transcriptomic profile obtained, we still were able to identify 41 candidate PEAGs among the CON vs. PTS groups. Gene ontology (GO) analysis indicated that the identified genes do not exhibit significant enrichment in any specific biological process, suggesting a functionally diverse transcriptional response. Given that functionally heterogeneous gene sets can still reveal regulatory complexity [95], we focused on genes with consistent expression patterns across PTS biological replicates, either uniformly upregulated or downregulated, and absent in unfertilized eggs, suggesting their paternal origin. This led us to identify 16 candidate PEAGs with shared expression patterns constituting valuable candidate PEAGs in Eurasian perch. Within this set we could identify genes involved in immune regulation, cell signaling, and developmental processes. Additionally, these genes exhibited notable patterns of differential expression, including both converging and diverging trajectories, across developmental stages. Such expression dynamics enhance the relevance of these genes as candidates for downstream validation and potential PEAGs.

The validation performed in this study shed light on several important immune-related genes. For instance, the *mfap4* gene which encodes a microfibril-associated protein that plays a role in organizing the extracellular matrix. In zebrafish, a common teleost model, *mfap4* is expressed in a subset of macrophages, making it a useful marker for these immune cells. This expression suggests that *mfap4* not only contributes to the structural integrity of tissues (for instance, in vascular and connective tissues) but may also be involved in innate immune responses and tissue remodeling during development [96]. Another noteworthy gene found is *gimap4*, which shows their higher expression in all the stages of the PTS group larvae, along with being absent in the UFE transcriptome. The GTPase domain of the immune-associated nucleotide binding protein (*gimap*) genes are rather huge family and are seen to be upregulated in response to infections [97, 98]. Another line of evidence of paternally-controlled expression of immune-related genes comes from the consistently high expression of major histocompatibility complex class I-related (*mhc*), also called as human leukocyte antigen (*hla-g*) gene, in the PTS group across the stages. Among many genes encompassed in the region, *hla-g* is a nonclassical class I molecule known to protect the progeny from maternal immune rejection [99]. However, functions of this gene are yet to be clearly understood in fish. We also found the continuous under-expression of the *pigr* gene in PTS group in Eurasian perch across larval period. The gene is again a part of the polymeric immunoglobulin receptor (*pigr*) superfamily and is said to be critically involved in IgM antibody-based local mucosal activity [100]. This, along with the other positively validated genes at MO stage, but compensated with age across other stages, include *mr1*, a paralog of *hla-g*; *neo1*, *neogenin*, which constitute a subgroup of the immunoglobulin superfamily [101] and are key components of the innate and adaptive immune system in vertebrates [102]. This contrasts with reports in cod (*Gadus morhua*), where immune transcripts are often maternally inherited [103], and suggests a potential paternal contribution to progeny immune system, which remains to be functionally validated. Additionally, the immune-related PEAGs identified at this stage may, like the observed early-life mortality, represent consequences of PTS rather than direct causes. However, the possibility that weakened immune function contributed to mortality cannot be excluded.

Among the validated candidate PEAGs, specific attention should be paid to *pde6g*. This gene produces the gamma subunit of the cyclic GMP-phosphodiesterase (pde6) enzyme which plays a critical role in regulating the enzyme’s activity, ensuring proper visual signal transmission and amplification [104]. This gene has already been reported in our previous experiments as putative PEAGs [27]. In the experimental setting, eggs were fertilized with either fresh or cryopreserved sperm, and upregulation of *pde6g* in progeny from cryopreserved sperm was observed. This finding suggested that sperm cryopreservation, by selecting for a subpopulation of sperm with distinct epigenetic (e.g., methylation) profiles, can reveal paternal-effect-associated genes, thereby influencing early eye development and potentially other sensory functions in the offspring. In the present study we found, where PTS became an additional selection pressure caused under-expression of this gene in the progeny. This indicates that while the paternal control over this gene is associated with positive cryo-selection, is negatively associated with further selection consequences during the PTS. Despite the exact mechanism needing to be further elucidated, our data constitute valuable additional evidence of paternal-control on expression of this gene.

## Conclusion

Our findings provide further evidence that paternal contributions, even when arising from subtle variations in sperm quality, can exert measurable effects on offspring phenotype and gene expression, functioning in concert with and under the modulation of maternal influences. We demonstrate that PTS of sperm acts as a dual selection pressure by eliminating sensitive spermatozoa while enriching sperm subpopulations that are more resilient. Beyond delivering the paternal genome, sperm carry regulatory RNAs, including mRNAs and microRNAs, that can modulate early embryonic development. Thus, selective filtering during PTS has the potential to alter progeny phenotype. Importantly, our previous results indicate that cryo-selection can serve as a positive filtering force [27], yet the associated storage-related stress imposes an additional layer of selection, with most likely detrimental consequences. Notably, both the higher early-life mortality and the predominance of immune-related PEAGs may be parallel consequences of PTS, though impaired immune function could still have contributed to mortality. Along with the transcriptomic data we also show the interplay between paternal effects and maternal modulation of gene expression which highlights the inherent complexity of parental contributions to developmental regulation. Finally, by integrating phenotypic assessments with transcriptomic analyses, we identified new candidate PEAGs and corroborated earlier reports of *pde6g* as a PEAG, thereby extending the current understanding of molecular mechanisms underlying paternal influences in fish reproduction.

It is important to acknowledge a limitation of our study, which is the use of a single, wild Eurasian perch population. Although informative, this design does not allow us to determine whether the phenotypic and transcriptomic consequences of PTS would manifest similarly in other perch populations, in domesticated broodstock, or even in other fish species with distinct reproductive physiologies. Such variation could arise from population-level and species-level differences in gamete characteristics, stress tolerance, and sensitivity to cryopreservation. Therefore, future studies incorporating domesticated stocks, multiple populations, and additional species will be essential to evaluate the generality and broader aquaculture relevance of the patterns reported here. Additionally, it should be emphasized that in our study we used an adjusted sperm-to-egg ratio, doubling the amount of sperm in the PTS group. This adjustment ensured that most of the eggs were successfully fertilized and developed further. However, it cannot be ruled out that using a similar and/or suboptimal number of spermatozoa for fertilization in both groups might have led to additional selection for different sperm cells, potentially resulting in a phenotype different from that observed in our study. Overall, these findings highlight promising directions for future research and demonstrate the robustness of this approach for investigating paternal effects in fish.

## Supplementary Information

Supplementary Material 1.

## Data Availability

Raw data from the analysis of different families of freshly hatched larvae can be accessed via the NCBI BioProject database under the PRJNA1265076 accession number.

## Abbreviations

ANOVA: Analysis of Variation
CASA: Computer–Assisted Sperm Assessment
CHR: Chromosome
CON: Control
CV: Coefficient of Variation
DEG: Differentially Expressed Gene
DNA: Deoxyribonucleic Acid
DPH: Days Post Hatch
LIN: Linearity
MO: Mouth Opening
MOT: Motility
NGI: Non–Genetic Inheritance
NIFRI: National Inland Fisheries Research Institute
PC: Principal Component
PCA: Principal Component Analysis
PEAG: Paternal–Effect–Associated Gene
PTS: Post Thaw Storage
RAS: Re–circulating Aquaculture System
RNA: Ribonucleic Acid
RT qPCR: Reverse Transcription Quantitative Polymerase Chain Reaction
SBIE: Swim Bladder Inflation Effectiveness
TL: Total Length
Tm: Melting Temperature
TPM: Transcripts Per Million
UFE: Un–Fertilized Egg
UNC: Uncharacterized
VAP: Average Path Velocity
VCL: Curvilinear Velocity
VSL: Straight–Line Velocity
WBW: Wet Body Weight
ZGA: Zygotic Genome Activation
